# Effective digital support for autism: digital social stories

**DOI:** 10.3389/fpsyt.2023.1272157

**Published:** 2024-01-03

**Authors:** Louis John Camilleri, Katie Maras, Mark Brosnan

**Affiliations:** Centre for Applied Autism Research (CAAR), University of Bath, Bath, United Kingdom

**Keywords:** autism, mobile application, digital intervention, social stories, large data

## Abstract

Social Stories™ is one of the most popular interventions for autistic children and has been researched extensively. However, effectiveness data has been gathered mainly through single-participant designs which generate outcomes which can lack generalizability and social validity. Stories Online For Autism (SOFA) is a digital application which supports the development and delivery of Social Stories in a real-world setting and has the potential to contribute toward furthering (1) Social Stories research and (2) research on digital applications for autism by gathering large data sets from multiple participants. Three data sets (*N* = 856) were gathered through the SOFA app and were analyzed to investigate three key variables: What predicted closeness-to-goal of the Social Stories (as rated by an adult/parent/guardian, *n* = 568); the child’s comprehension of the Social Stories (assessed by story comprehension questions, *n* = 127); and the child’s rating of the enjoyability of the Social Stories (*n* = 161). A merged data set then investigated correlations between these three key variables. Age range (≤15), gender, autism diagnosis, and the child’s level of language understanding were the potential predictors for these three key variables. Regression analysis indicated that parental closeness-to-goal ratings for their children were highest for children who were younger and more verbal. Regression analysis also indicated that older children scored higher in comprehension assessment, and autistic children rated the Social Stories as more enjoyable. Closeness-to-goal, comprehension scores and enjoyment ratings did not significantly correlate with each other. This is the largest study of Social Stories effectiveness, which was enabled through the collection of data through a digital app from multiple participants. The results indicate that digital social stories are particularly effective for younger verbal children. While this was the case for all children, it was particularly true for autistic children and female (and gender-diverse) children. For the first time, the gathering of large digital data sets has highlighted that while digital Social Stories can be effective for autistic males, they can be more effective for autistic females and gender-diverse autistic individuals. Thus, the SOFA app can support the investigation of the factors which influence Social Stories outcomes that are generalizable and with high social validity.

## Introduction

Autism spectrum disorder (hereafter autism) is a neurodevelopmental condition characterized by differences in social communication and social interaction and the presence of restricted, repetitive behaviors (1). Digital platforms are being increasingly utilized to support the development and delivery of interventions and support for autistic individuals (2, 3). Reviews of literature [e.g., (4, 5)], highlight the use of technology-based interventions for autistic children which are delivered through desktop computers, interactive DVD, shared active surfaces (i.e., applications designed for simultaneous, in-person collaboration among multiple users, such as expansive screens and electronic whiteboards), and virtual reality. Technology tools develop and change swiftly, as do the expectations from the contexts of their use (6). One of the most recent applications of technology within the autism community is digital technology in the form of smartphone applications (7). Since the launch of the Apple Store in 2007 and Google Store in 2012, together with the increase in the availability of hand-held smart-phone technology, digital applications have increasingly been utilized to support autistic individuals in their academic learning (8, 9), to improve their cognitive skills (10), and to improve socio-adaptive behaviors (11, 12). By 2018 nearly 700 “autism apps” for smartphones and tablets were available (13), highlighting an increase in interest and awareness of the potential of digital smartphone applications to support the autism community. The recent increase in interest in digital technology supports for autism is argued to reflect advantages related to enabling caregiver (or self) delivered support (14), creating structured and predictable environments (15), enhancing visual support, self-monitoring, and rewards (16, 17) as well as facilitating repetition and direct feedback (18).

Notwithstanding such an increase in popularity, there is a dearth of research on the effectiveness of digital applications and their utility to support the autistic community. Kim et al.’s (13) review indicates that only around 10% of existing autism apps have available evidence which goes beyond the anecdotal kind. Furthermore, a meta-analysis by Sandgreen et al. (19) on the use of digital interventions (including digital apps) to support autistic individuals highlighted a small effect size as well as a high heterogeneity between studies that were reviewed. Thus, the authors recommended that future studies include larger data sets which can improve the external validity of studies with the aim of generalizing results to other populations, settings, and times beyond the specific sample and conditions studied. The Social Stories™ intervention developed for autistic children is an ideal example to explore the impact of digitalization upon effectiveness data. Social Stories was introduced by Gray and Garand (20) and is one of the most widely used interventions for autistic children (21, 22).

The Social Stories intervention consists of narratives that are aimed toward supporting the transfer of information between the authors of the stories and the audience (23), which are typically autistic children and adolescents, though non-autistic children can also benefit from the intervention (24, 25). The development of a story starts by identifying a title and a goal. Titles and goals are distinct entities: while the title serves to identify the topic and establish the context for the story’s content, the goal represents the specific intention that the story seeks to achieve. Some examples of titles are “Taking the temperature” (see Appendix A for an example of this social story) or “Taking a bath.” The goals for these respective stories are “To increase the child’s understanding of how a doctor uses a thermometer to measure body temperature,” and “To prepare the child on what to expect when using a bath to bathe.” The narratives are delivered in text format and are written in a positive tone. They contain precise and detailed content that accurately depicts a specific subject, experience, or phenomenon. The length of each story, i.e., the number of sentences and phrases, can vary based on the individual’s needs and characteristics. The narratives may also incorporate images or pictures that complement or accentuate the text.

The initial guidelines proposed by Gray and Garand (20) highlighted the importance of crafting stories from the child’s perspective. Over time, guidelines related to the style and format of the stories have evolved (23). Presently, the guidelines for composing Social Stories have advanced to their third version and consist of a set of ten criteria which aim to explain the process of developing the story as well as the story’s structure (26). The first of Gray’s criteria sets the tone of the intervention and highlights the need for authors to engage with the audience’s experience and perspective of the world around them. The second criterion encourages the process of identifying relevant information about the audience and about the specific topic of the Social Story. The third criterion outlines the composition of each story. This involves providing a distinct title and ensuring that every story comprises an introduction that outlines the topic, a body that elaborates on details, and a conclusion that reinforces the information. The third criterion also defines the two types of sentences which stories should involve. Gray employs the term “Descriptive Sentences,” which accurately portrays the relevant aspect of a context without passing judgment. The term “Coaching Sentences” is utilized to denote sentences that gently guide the audience. The fourth criterion details that each story should be adjusted to align with the audience’s attention span, learning style, and interests. The fifth criterion emphasizes the need for stories to adopt a patient and supportive tone, advocating for their composition in either the first or third person. The sixth criterion outlines that a story should address pertinent ‘who,’ ‘what,’ ‘when,’ ‘where,’ ‘why,’ and ‘how’ questions. The seventh criterion specifies that 50% of stories should be crafted for a specific individual, aiming to celebrate and praise. The eighth criterion dictates that each story should include three times as many descriptive sentences as coaching sentences. Criteria 9 and 10 underscore the iterative process of refining and enhancing the story. The intervention has been utilized frequently to support autistic individuals in tasks such as the reduction of inappropriate behaviors, improvement in social behaviors, supporting the acquisition of academic and functional skills, and assisting in novel events/transitions (27).

Social Stories research is extensive (28–30) but comprises a body of research which is mostly informed by single-case experimental designs (31). These research designs can lead toward limited generalizability, and result in outcomes which are not necessarily applicable to populations that go beyond the characteristics of individual participants from the single-case studies, which in turn can impact the highly variable outcomes reported in Social Stories research (32). The Stories Online for Autism (SOFA) application is a digital application, for smartphones and tablets, which can support the development and delivery of the Social Stories intervention in real-world settings and enable the collection of large data sets from multiple participants.

The SOFA app is an application that can be freely downloaded from the Apple Store and Google Play Store and can be installed on iOS and Android devices. The application has the potential to support the digital delivery of Social Stories through a number of features (see Appendix B) aimed toward increasing procedural integrity (i.e., the extent to which an intervention is implemented as intended by the developers) by focusing on goal-setting, goal-rating, and goal-monitoring. The app provides features for the personalization of story delivery (see Appendix C). Furthermore, the application can support the gathering of large data sets in real-world settings (i.e., in contexts that go beyond clinical studies). Thus, the application can be utilized to mitigate the issue of variability in existing Social Stories outcome research while also seeing to the issue of external validity.

The application was developed together with the autistic community and utilized a participatory design informed by the Interface Design Experience for the Autistic Spectrum (IDEAS) framework (33, 34). The SOFA app can support the development and delivery of Social Stories by operating in two modes: The adult (writing) mode and the child (reading mode). The writing mode is where authors develop their stories by using a well-defined, intuitive, and user-friendly writing interface. The writing interface supports the author to write appropriately structured Social Stories (see Appendix D). The author also rates the extent to which the audience has reached the goal of the Social Story (i.e., child’s closeness-to-goal) on an 11-point (0 to 10) Likert Scale. The reading mode is where individuals may access, view and/or read stories, and includes a text-to-speech function (i.e., a function which converts written text into an audio output). The SOFA app includes a fill-in-the-blank comprehension check at the end of each story that has been read. The SOFA app also includes a feature that assesses story enjoyment from the audience’s perspective. Thus, three key variables are evaluated through the digitalization of the Social Stories intervention.

The SOFA app provides the opportunity to easily tailor a Social Story for the audience, and in so doing encourages further engagement with the audience’s (i.e., the child’s) perspective. This is also achieved by using photos that are taken by the author on their smartphone which are stored in the device’s image gallery. This feature enables authors to include real images of people, objects, and settings in their social stories. The application’s social story library can be used to store and share stories with the SOFA-users community. In this manner, the autistic and broader autism communities are encouraged to share their Social Stories and thus collaborate indirectly with other users. The application also allows users to download stories in PDF formats. The SOFA app has been utilized to investigate how the digital mediation of the Social Stories intervention can support autistic children in adapting to change (35), and to increase understanding and reduce anxiety (36). The SOFA app has also been reported to impact positively procedural integrity while also empowering end users (37, 38). Thus, the SOFA app has the potential to support the development and delivery of Social Stories. Furthermore, given the wide appeal of digital distribution platforms for mobile applications, the SOFA app can be used to gather large data sets which can help answer questions related to the effectiveness of digital supports for the autistic community, while also contributing toward generalizable and socially valid Social Stories research.

For the first time, this study utilized “large data” gathered through the SOFA app to investigate the potential of digital technology to mediate the Social Stories intervention. In this case, the term “large data” is defined as a dataset that is “larger than what researchers conventionally handle in their respective fields” [(39), p. 2]. The application was downloaded 1,000+ times and allowed for the gathering of large anonymized data sets which can contribute further toward the investigation of digital apps as well as to Social Stories literature.

## Methods

### Research aims

The study aimed to utilize the data gathered from the SOFA app to investigate the following questions:

1. What factors predict story closeness-to-goal ratings?
2. What factors predict story comprehension?
3. What factors predict the child’s story enjoyability rating?
4. Is there an association between adult closeness-to-goal rating, comprehension, and enjoyability rating?

### Data collection

This study used anonymized data gathered through the SOFA app research project^1^ which is led by the University of Bath’s Centre for Applied Autism Research (CAAR). Participants who downloaded the app were informed that the SOFA app collects data on how effective the stories are in helping autistic children achieve their goals. To achieve this, the SOFA application gathered data about (1) the title of the story used, (2) the child’s communication level (Pictures only, single words, simple sentences, and full sentences) (3) the child’s age group (Under 4 years, 5 to 10 years, and 11 to 15 years), (4) the child’s gender (Male, female, other), and (5) the child’s diagnosis (i.e., autistic or non-autistic) (18, 35, 36).

### Data sets

Three data sets were collected through the SOFA app. The first data set consisted of data about the child’s closeness-to-goal, as rated by the authors of the story (i.e., the parents or guardians). Closeness-to-goal refers to how near or far a child is to achieving a pre-determined objective or target. It was measured on an 11-point Likert Scale that was utilized to allow for flexibility and personalization of the scaling process (18, 40). A value of 0 indicates that the goal is not at all reached, 5 indicates that the goal is halfway toward being reached, and 10 indicates that the goal is completely reached.

The second data set consisted of data about the child’s understanding of the story as measured by three comprehension questions. After a story has been read, the child is presented with three questions in the form of fill-in-the-blank. These questions are automatically computed by the SOFA app and are based on the sentences which are used in the story. The children are provided with a choice of three potential answers to the questions, from which they are invited to choose one. The instructions (i.e., to “Choose the right missing word”), the fill-in-the-blank sentence, as well as the three potential answers can be read out to the user through text-to-speech functionality.

The third data set consisted of the child’s rating of the enjoyability of Social Stories. After every story which is read, the child is asked to rate the story. This is done by inviting the child to answer the question “What did you think of the story?” The child is then presented with five potential replies: Brilliant, really good, good, not good, and awful. The replies are accompanied by corresponding visuals in the form of smiley emojis which they can select from.

A final merged data set consisted of an amalgamation of the first three data sets. I.e., story titles and corresponding child characteristics across the three data sets were identified. This allowed for the comparison of data across three data sets.

### The participants

The participants were all parents/guardians of children, or adults supporting children. Most of the children were described as autistic while some were described as non-autistic. All the participants downloaded the application onto their digital devices and utilized the app at their discretion. Each participant used the application to (1) create an “author” (or adult) account which enabled them to develop Social Stories, (2) create an “audience” (or child) account which enabled them to create a profile for their child, and (3) create stories through the adult account and assign them to the specific child account for the child to be able to read the story on their digital device. The characteristics of the children who were reading the stories are summarized in Table 1.

**Table 1:** Participant characteristics.

	Data sets					
	1		2		3	
	Frequency	Percentage	Frequency	Percentage	Frequency	Percentage
*N*	568	100	127	100	161	100
Gender						
Male	401	70	92	72	111	69
Female	135	24	24	19	33	21
Other	32	6	11	9	17	10
Communication level						
Pictures only	35	6	-	-	-	-
Single words	64	11	16	13	22	14
Simple sentences	225	40	46	36	59	37
Full sentences	244	43	65	51	80	49
Age range (years)						
Under 4	126	22	34	27	44	27
5 to 10	328	58	69	54	87	54
11 to 15	114	20	24	19	30	19
Diagnosis						
Not autistic	107	19	25	20	34	21
Autistic	461	81	102	80	127	79

Data set 1 = Adult’s closeness-to-goal ratings; Data set 2 = Comprehension questions; Data set 3 = Child’s story rating.

### Analysis

#### Data set 1

Data set 1 consisted of closeness-to-goal rating given by adults. These ratings are indicative of how close the adults rate their children to be in reaching the goals which have been set. The ratings were given on a Likert scale ranging from 0 (goal not met) to 10 (goal completely met).

The original data set consisted of 794 data points. Each point consisted of a closeness-to-goal rating which was given by an adult in relation to their child’s closeness-to-goal. Some stories were rated multiple times. Each child was rated on average for two stories. Each story was rated from one to nine times (i.e., the same story could have been rated multiple times at different occasions). Thus, the data was “cleaned” by averaging the ratings of stories which were rated for the same user. In this way, the data points were reduced to 568 where each data point was independent of each other. Thus, when the same story was rated multiple times for the same child, these ratings were averaged.

Other data that was gathered was (1) the child’s gender (male, female, and other), (2) if the child was autistic or not, (3) the child’s communication level (Pictures only, single words, simple sentences, and full sentences), and (4) the child’s age range (under 4 years, 5 to 10 years, and 11 to 15 years).

A multiple regression was run to predict closeness-to-goal rating from gender, age, communication level and autism diagnosis (i.e., autistic vs. non-autistic). There was independence of residuals, as assessed by a Durbin-Watson statistic of 1.817. There was homoscedasticity, as assessed by visual inspection of a plot of studentized residuals versus unstandardized predicted values. There was no evidence of multicollinearity, as assessed by tolerance values greater than 0.1. The assumption of normality was met, as assessed by a Q-Q Plot.

#### Data set 2

After reading the story on SOFA app, a comprehension check was presented to the user through 2 to 3 questions. The data set on the results of the comprehension scores consisted of 552 data points. Each point consisted of a story comprehension score which the audience (i.e., the child) completed for each story they read.

Some of the children completed a comprehension task for the same story multiple times. Thus, this meant that every data point was not independent of each other. For this reason, instances where the same child completed a comprehension check for the same story have been averaged. This resulted in 127 data points where each data point is the average comprehension score of a child on a specific story. The dependent variable in this data set consisted of an ordinal variable with three categories of comprehension scores: none correct, some correct, all correct.

An ordinal regression was run to determine the effect of gender (male, female and other), age group (0–4, 5–10, 11–15 years), autism diagnosis (not autistic, autistic), and level of language understanding (full sentences, simple sentences, single words^2^) on the user’s comprehension scores (none correct, some correct, all correct).

The deviance goodness-of-fit test indicated that the model was a good fit to the observed data, *χ*^2^(39) = 36.992, *p* = 0.562. The Pearson goodness-of-fit test also indicated that the model was a good fit to the observed data, *χ*^2^(39) = 33.951, *p* = 0.699. However, most cells were sparse with zero frequencies in 33.3% of cells. The final model did not significantly predict the dependent variable over and above the intercept-only model, *χ*^2^(7) = 4.275, *p* = 0.748, *R*^2^_CS_ = 0.097, *R*^2^_N_ = 0.115.

#### Data set 3

Data was gathered on enjoyability ratings given by children who read the story. These ratings were indicative of how much the children enjoyed the story (Brilliant, really good, good, not good, and awful).

The original data set consisted of 695 data points. Each point represented a story rating given by a child after reading a particular story on the SOFA app. Some of the children rated the same story multiple times. Thus, this meant that every data point was not independent of each other. For this reason, instances where the same child rated the same story were averaged. This resulted in 161 data points where each data point was the average story rating by a child for a specific story.

An ordinal regression was run to determine the effect of gender (male, female and other), age group (0–4, 5–10, 11–15), autism diagnosis (autistic, not autistic), and language understanding level (full sentences, simple sentences, single words^2^) on the story enjoyability rating given by participants (Brilliant, really good, good, not good, and awful). The deviance goodness-of-fit test indicated that the model was a good fit to the observed data, *χ*^2^(117) = 112.860, *p* = 0.591. Again, in this data set, most cells were sparse, with zero frequencies in 51.9% of cells. However, the final model statistically significantly predicted story rating over and above the intercept-only model, *χ*^2^(117) = 22.342, *p* = 0.002. *R*^2^_CS_ = 0.130, *R*^2^_N_ = 0.137. For this model, the proportional odds assumption appears to have held because the Chi-Square statistic was not significant, *p* = 0.227.

#### Merged data set

The three data sets were merged and data points were matched. A Spearman’s rank-order correlation was run to assess the relationship between adult closeness-to-goal rating, comprehension and child story rating.

### Ethics statement

This study received ethical approval from the University of Bath Psychology Research Ethics Committee (PREC, Project ID 19–309).

## Results

### What predicts adult closeness-to-goal rating (Data set 1)?

The multiple regression model (with gender, age, communication level and autism diagnosis as independent variables) statistically significantly predicted closeness-to-goal rating, *F*(8, 559) = 5.891, *p* < 0.001. This four-predictor model has an R^2^ of 7.8% with an adjusted R^2^ of 6.5%. Regression coefficients and standard errors can be found in Table 2.

**Table 2:** Multiple regression for closeness-to-goal rating (Data set 1).

Closeness-to-goal Rating	*B*	95% CI for *B*		SE *B*	β	R^2^	ΔR^2^
		LL	UL				
Model						0.078	0.065
Constant	4.314^**^	3.611	5.017	0.358			
Diagnosis							
Non-autistic	−0.732^*^	−1.311	0.153	0.295	−0.103^*^		
Gender							
Other	1.240^*^	0.262	2.217	0.498	0.103^*^		
Female	0.917^**^	0.386	1.448	0.270	0.141^**^		
Communication level							
Pictures only	−1.712^**^	−2.681	−0.744	0.493	−0.148^**^		
Single words	0.400	−0.366	1.165	0.390	0.046		
Simple sentences	0.460	−0.038	0.957	0.081	0.081		
Age range							
Under 4 years	0.823^*^	0.115	1.531	0.123	0.123^*^		
5 to 10 years	0.749^*^	0.168408	1.330	0.133	0.133^*^		

Model, “Enter” method in SPSS Statistics; B, unstandardized regression coefficient; CI, confidence interval; LL, lower limit; UL, upper limit; SE B, standard error of the coefficient; *β*, standardized coefficient; R^2^, coefficient of determination; ΔR^2^, adjusted R^2^. *p < 0.05, **p < 0.001.

Autistic children scored, on average, 0.732 higher than non-autistic children in their closeness to goal ratings (when other effects were kept constant). Females received scores 0.917 higher than males, while children whose gender was listed as “other” received scores 1.240 higher than males. Minimally verbal children (i.e., whose communication level was described as pictures only) received scores which were 1.712 lower than children who used full sentences to communicate. Younger children (under 4 years of age) received scores 0.823 higher than older children.

In summary, adults’ closeness to goal ratings were highest for autistic children who were younger, verbal, and not male.

### What predicts comprehension (Data set 2)?

Analysis of parameter estimates indicate that the child’s gender, communication level and diagnostic category were not significant predictors of comprehension scores. However, parameters indicated that younger children (under 4 years) were more likely to obtain a lower comprehension score than the older children (11 to 15 years), Odds Ratio = −1.593 (95% CI, −2.762, −0.424), *χ*^2^(1) = 7.127, *p* = 0.008. In summary, comprehension scores were highest for older children. Odds ratios and value of *p*s can be found in Table 3.

**Table 3:** Ordinal regression for results of the comprehension scores (Data set 2).

Variable	Odds ratio	SE	95% CI Lower, Upper	Wald *χ*^2^	Value of *p*
Age group					
Under 4	−1.593	0.597	−2.762, −0.424	7.127	0.008
5 to 19 years	−0.758	0.510	−1.759, 0.242	2.203	0.137
Diagnosis					
Non-autistic	0.199	0.482	−0.746, 1.144	0.171	0.680
Communication level					
Single words	−0.180	0.595	−1.347, 0.987	0.091	0.763
Simple sentences	−0.311	0.408	−1.111, 0.488	0.582	0.446
Gender					
Other	−1.086	0.662	−2.383, 0.212	2.689	0.101
Female	0.674	0.499	−0.303, 1.651	1.828	0.176

SE, Standard Error; CI, Confidence Interval.

### What predicts the child’s enjoyability story rating (Data set 3)?

The odds ratio of obtaining a higher story enjoyability rating for autistic children was 1.044, 95% CI [0.258, 1.831] times that of non-autistic children, a statistically significant effect, *χ*^2^(1) = 6.771, *p* = 0.009. Odds ratios and value of *p*s can be found in Table 4. Taken together, the stories were rated highest by autistic children.

**Table 4:** Ordinal regression for results of child’s story ratings (Data set 3).

Variable	Odds ratio	SE	95% CI Lower, Upper	Wald *χ*^2^	Value of *p*
Age group					
Under 4	−0.202	0.464	−1.112, 0.708	0.189	0.663
5 to 19 years	0.622	0.396	−0.154, 1.397	2.469	0.116
Diagnosis					
Non-autistic	−1.044	0.401	−1.831, −0.258	6.771	0.009
Comm					
Single words	0.760	0.478	−0.177, 1.698	2.525	0.112
Simple sentences	0.279	0.340	−0.387, 0.946	0.674	0.412
Gender					
Other	0.172	0.490	−0.788, 1.132	0.123	0.726
Female	0.037	0.380	−0.708, 0.781	0.009	0.923

SE, Standard Error; CI, Confidence Interval.

### Is there an association between adult closeness-to-goal rating, comprehension, and enjoyability rating?

There was no statistically significant correlation between the variables (all *r*_s_ < 0.13, all *p*_s_ > 0.15, see Table 5). Correlation coefficients and value of *p*s can be found in Table 5.

**Table 5:** Spearman’s rank-order correlation.

Measure		1.	2.
Adult rating		-	
Comprehension	Correlation coefficient	−0.090	–
	Value of *p*	0.315	
	*N*	128	
Child’s story enjoyability rating	Correlation coefficient	0.098	0.126
	Value of *p*	0.224	0.158
	*N*	157	128

## Discussion

The SOFA application has great potential for users (i.e., story authors and audiences) as it provides user-friendly, portable, and structured support for the development and delivery of the Social Stories intervention. It also presents with potential for research; specifically with data collection. The digital format of the intervention has enabled the analysis of 856 data points. To our knowledge, this constitutes the largest analysis of the social stories intervention, facilitated by internet-based data collection within the SOFA app to date. Three key variables were evaluated: closeness to goal ratings, story comprehension and story enjoyment.

The results from the adults’ closeness-to-goal data set (data set 1) indicate that ratings were highest for autistic children who were younger, verbal, and not male. This data set suggests that Social Stories are particularly effective for autistic children (compared to non-autistic children). Social Stories were also more effective for primary school-aged children (aged under 11) when compared to secondary school-aged children (aged 11–15). Furthermore, Social Stories were more effective when the children were verbal, whether the language level was single words, simple or full sentences – when compared to children who were using pictures to communicate. Taken together, the analysis of this large data set (*n* = 568) has highlighted that Social Stories are most effective for autistic, verbal, primary-school-aged children. This is consistent with previous research which indicates that Social Stories are effective for autistic school-aged children (24) and children with “higher” verbal skills (41, 42). It may be the case that Social Stories are particularly effective for autistic females, which can be explored in future research. This may also be the case for those who identify as gender-diverse, who are over-represented in autistic populations (43, 44). However, the analysis indicated that the model used to predict adult closeness-to-goal ratings, while producing a statistically significant result, only explained 7.8% of the variability (6.5% adjusted). Thus, this suggests that other factors besides gender, communication level, age, and diagnosis are influencing closeness-to-goal outcomes. Again, future research using large data sets can explore what these other factors might be and may include factors such as what behavior the story is addressing (32).

The results of the child’s comprehension score (data set 2, *n* = 127) indicate that children who are under 4 years of age obtained the lowest comprehension scores when compared to older children. Thus, younger children reported a poorer understanding of the stories which they read. This is in line with previous research which reports that the child’s comprehension skills are a factor that impacts Social Stories effectiveness (42, 45). The results from the child’s story enjoyability rating data set (data set 3, *n* = 161) indicate that the stories were rated highest for enjoyability by autistic children when compared to non-autistic children. This may be because autistic children particularly appreciate (and potentially benefit from) the explicit structure of the stories (23).

It was interesting that the three key variables (Table 5) did not correlate with each other, suggesting that closeness to goal ratings were not significantly related to the child’s comprehension or enjoyment of the stories. Thus, the question of the importance of enjoyability and its association with intervention outcomes could be explored further in future research. I.e., is “enjoyability” directly related to Social Stories outcomes or is it only important to ensure commitment toward the intervention procedure? Furthermore, it is also important to keep in mind that closeness-to-goal, as rated through an adult lens, might not necessarily equate directly to the success of the intervention. A compelling argument can be made for considering a child’s comprehension scores as a crucial element in evaluating intervention success. Higher comprehension scores signify a grasp of the story and, consequently, a successful transfer of information through the Social Stories intervention, aligning with Gray’s (46) assertion that information transfer is the primary objective of Social Stories. However, it’s important to acknowledge that adult authors’ closeness-to-goal ratings provide a reasonable and sound summary of behavioral and social outcomes, as demonstrated in previous Social Story research [see (40)]. Yet, these ratings may be subject to various influencing factors, including goal clarity and adult expectations, which can affect how success is perceived. Therefore, while closeness-to-goal ratings offer a useful snapshot of intervention success, a nuanced understanding considers additional factors in evaluating the overall effectiveness of the intervention.

## Limitations

The 1,000+ downloads, as reported on both Google Android Store and Apple’s Appstore are indicative of a high interest in digital applications and also confirm the popularity of the Social Stories intervention. Shic and Goodwin (3) argue that portability, increased sophistication, and ubiquity are factors which could be popularizing digital applications as support tools for autistic individuals. One could speculate on the reason for the popularity of digital technologies such as the SOFA app. This could be related to the ease of access and availability of such applications. The prospect and promise of digital support in the development of Social Stories could also be a reason for the high number of downloads. Thus, it could be difficult to separate the technology from the intervention (i.e., the SOFA app from the Social Stories intervention), and as a consequence, it may be difficult to identify if the technology is what is spurring the interest in the app, or if it is the Social Stories intervention *per se*. The same can be argued about the outcomes of the intervention, i.e., it is difficult to attribute outcomes to anything other than both processes (Social Stories and digital platform) equally. To address such issues, future research would benefit from employing randomized control trials (RCTs) or quasi-experimental designs with control conditions to ascertain which component of the intervention impacts the observed outcomes. Such designs would improve internal validity (e.g., by minimizing the influence of confounding variables). Moreover, survey-based or qualitative investigations may provide additional insights into the factors motivating interest in the SOFA app, specifically discerning whether it is the digital dimension of the intervention or the Social Stories themselves that serve as the primary catalyst.

Additionally, there is no current existing data that compares the actual achievement of children’s goals with the perceptions of adults, particularly as reflected in their closeness-to-goal ratings. While the closeness-to-goal measure is commonly employed in intervention outcome research, its validity as a sole indicator of intervention success may be questionable. Enhanced validity could be achieved if the closeness-to-goal ratings provided by adults could be substantiated by independent raters. This approach has the potential to offer a more robust understanding of the intervention outcomes. However, implementing such a strategy might pose logistical challenges, potentially reducing the amount of data gathered and also conflicting with the intended intervention aims, which are to facilitate the transfer of information while concurrently assisting individuals in achieving goals deemed significant for the target audiences. Nevertheless, the inclusion of personalized outcome measures, such as Goal Attainment Scaling (47) or Goal-Based Outcome frameworks (48), which involve selecting and scaling goals to evaluate the degree of goal achievement for individuals, can be seen as beneficial in supporting similar research designs.

The SOFA app invites users to input data in all available fields. However, one cannot ascertain that the data which has been inputted is necessarily accurate, as it is all self-report. This is especially important for data pertaining to the gender and diagnosis categories. Users are also encouraged to utilize all of the features made available on the SOFA app. This includes inviting the children to rate the stories after reading, and also complete the comprehension check. It also includes inputting the adult closeness-to-goal rating. A prompt (through a visual reminder) is sent to the adult, through the application, to complete the closeness-to-goal rating for each story. Thus, adults can rate the story each time a story has been delivered (i.e., each time a child has read the story). However, the responsibility lies with the adult to submit the closeness-to-goal ratings. If users choose not to utilize all of SOFA’s available features, this could impact the quality and quantity of data which is gathered, while potentially compromising the intervention’s procedural integrity. In the data sets, it was evident that not all the features of the application were utilized, and thus, this impacted the quantity of the data points gathered in these datasets. To address such limitations, future research could utilize multiple data sources or methods (such as observations) to enhance the reliability and validity of data.

Furthermore, future research should also seek confirmation of participants’ clinical diagnoses of autism and gather further comprehensive clinical information via assessments administered by qualified clinicians. This information should encompass aspects such as the severity of clinical symptomatology and the assessment of cognitive functioning ascertained through the judicious utilization of assessments administered by qualified clinicians, thereby transcending an exclusive reliance on self-report measures while also addressing data reliability concerns.

Finally, an analysis of the composition of the actual stories was not carried out in this study. Future research endeavors could consider investigating the composition of developed stories through SOFA, thereby contributing to the exploration of the significance of Gray’s criteria in the development of Social Stories. Such investigation also holds the potential to enhance the existing literature on the theoretical foundations of the Social Stories intervention, an area that currently lacks thorough examination (23).

## Conclusion

The SOFA app is a digital application for smartphones and tablets which supports the digital mediation of the Social Stories intervention. The app aims to support story authors with the development and delivery of stories. In this study, the application was used to gather the largest data set on Social Stories to date and explore the factors that could relate to the effectiveness of the Social Stories intervention. The analysis of the data gathered confirms the importance of autism diagnosis, age, gender and language skills for the Social Stories intervention while also confirming the usefulness and appeal of the Social Stories intervention, as well as the appeal of digital apps, to the autistic and broader autism communities. This study also highlights the usefulness of digital applications for research as they can provide opportunities for the collection of large data sets which can mitigate the issue of heterogeneity of Social Stories research.

## Data availability statement

The raw data supporting the conclusions of this article will be made available by the authors, without undue reservation.

## Ethics statement

The studies involving humans were approved by University of Bath’s Psychology Research Ethics Committee (PREC, Project ID 19-309). The studies were conducted in accordance with the local legislation and institutional requirements. Written informed consent for participation was not required from the participants or the participants’ legal guardians/next of kin. However, all the participants who downloaded the app (https://sofa-app.org) were informed that the SOFA app collects data on how effective the stories are in helping autistic children achieve their goals. The users of the application agreed to the following terms and conditions upon registering their account: “As part of this research process, the SOFA-app collects data on how effective the stories are in helping autistic children achieve their goals. All data is securely stored in compliance with GDPR legislation. By registering and submitting your personal details you consent to the University of Bath using your details for administering its database of stories and contributors for the purposes of this project, including managing your account (forgotten passwords etc)”.

## Author contributions

LC: Data curation, Formal analysis, Investigation, Methodology, Writing – original draft. KM: Supervision, Writing – review & editing. MB: Supervision, Writing – review & editing.

## Footnotes

^1^https://SOFA-app.org

^2^The “language understanding level” originally consisted of 4 categories. However, for this data set, only 2 users informed the “pictures only” category. Thus, this category was removed from the analysis.
