# Supplementary material for: Effective digital support for autism: digital social stories
Source: Front Psychiatry. 2024 Jan 3;14:1272157. doi: 10.3389/fpsyt.2023.1272157 (PMC10791792; doi:10.3389/fpsyt.2023.1272157)
Supplement: Supplementary file 1 [file Data_Sheet_1.docx]

**Appendix A –** *A script for a social story.*

**Title of story:** Taking my temperature

**Goal:** To increase the child’s understanding of how a doctor uses a thermometer to measure body temperature.

**Story text:**

Sometimes if I am sick, I get very hot.

I might need to go see the doctor.

The doctor might take my temperature with a thermometer to find out how hot I am.

He might ask me to put a thermometer in my mouth, under my tongue.

He might put a thermometer in my ear. This will not hurt.

I might hear a beep or a click noise, and this will not hurt.

The doctor can see if I am sick.

The doctor can make me feel better.

Going to the doctor is ok.

**Link to view the story as presented on SOFA:** <https://www.youtube.com/watch?v=uBSmrrwvy3o>

**Appendix B -** Features of the SOFA app’s Adult (Writing) Mode

| **Feature** | **Description** |
| --- | --- |
| Create good social stories. | A video “walk-through” is available as soon as the adult account is first opened. This walkthrough guides authors on how to write good stories. |
| Find support on how to write the best stories. | An extensive help section is available for authors on the criteria required to write good stories. |
| Create multiple child profiles. | A profile may be created for each child. That way a story, or a different version of a story, may be assigned to a particular child. |
| Easily incorporate images in the stories. | This is done by utilising real pictures taken from the device’s digital camera. Users can also choose images from an image library. |
| Monitor progress towards story goals. | This is achieved through goal setting Likert scales and prompts for monitoring after reading of every story. |
| Develop personalised stories. | This is achieved through the “Create a story” feature, which allows personalisation of the story goal and story text. Each story can be edited as many times as needed. |
| Use story templates. | Story templates are found in the story library, where stories which have been set as “public” may be edited to fit an individual’s specific needs. |
| Use personal photos and stories privately. | Each story, as well as each image used in a story, may be stored privately in an individual’s account, or publicly in the story library. |
| Personalise the reading layout for your audience. | The app allows for three reading layouts for the stories: 1) book story format, stacked pictures, and text only. The authors may also decide to select the “Let my child decide” function, which will then ask the child which layout they want to use before they read a story. |
| Export story to PDF | Each story may be downloaded to PDF format. |

**Appendix C** – Features of the SOFA app’s Child (Reading) Mode

| **Feature** | **Description** |
| --- | --- |
| Select which story to read. | The audience (i.e., the child) may independently select which story assigned to that child they want to read. |
| Select in which format the story is presented. | The audience may choose which format the story is presented (i.e., book story format, stacked pictures, and text only.) |
| Have the story read out to you. | The text-to-speech function on the SOFA app allows for all the text on each screen to be read out for the audience. |
| Rate story enjoyment. | After each story is read, the audience is invited to rate how much the enjoyed the story. |
| Check for understanding. | This is done by answering fill-in-the blank multiple-choice questions at the end of reading each story. |
| Personalise the screen. | The audience may decide to personalise the screen by changing their profile’s colour scheme. |

**Appendix D** – Images of the SOFA Home Screen and Adult (Writing) mode.


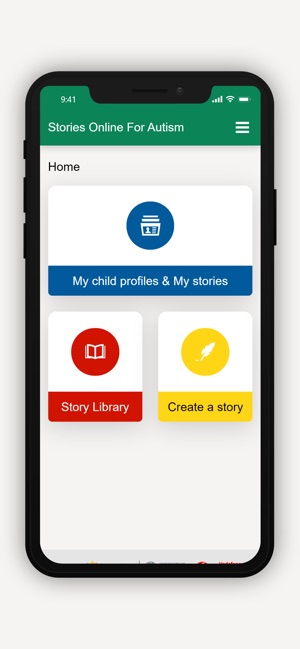

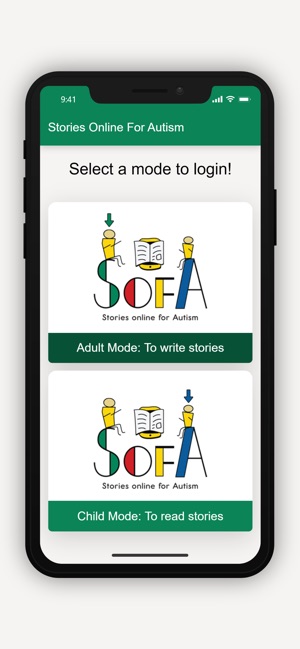


**SOFA Adult (writing) Mode**

**SOFA Home Screen**
